# The epidemiology of polyparasitism and implications for morbidity in two rural communities of Côte d’Ivoire

**DOI:** 10.1186/1756-3305-7-81

**Published:** 2014-02-25

**Authors:** Eveline Hürlimann, Richard B Yapi, Clarisse A Houngbedji, Thomas Schmidlin, Bernadette A Kouadio, Kigbafori D Silué, Mamadou Ouattara, Eliézer K N’Goran, Jürg Utzinger, Giovanna Raso

**Affiliations:** 1 Department of Epidemiology and Public Health, Swiss Tropical and Public Health Institute, P.O. Box, CH-4002, Basel, Switzerland; 2 University of Basel, Basel, Switzerland; 3 Département Environnement et Santé, Centre Suisse de Recherches Scientifiques en Côte d’Ivoire, Abidjan, Côte d’Ivoire; 4 Unité de Formation et de Recherche Biosciences, Université Félix Houphouët-Boigny, Abidjan, Côte d’Ivoire; 5 Unité de Formation et de Recherche Sciences de la Nature, Université Nangui Abrogoua, Abidjan, Côte d’Ivoire; 6 Unité de Formation et de Recherche Communication, Milieu et Société, Université Allassane Ouattara, Bouaké, Côte d’Ivoire

**Keywords:** Anaemia, Côte d’Ivoire, Helminth, Malnutrition, Morbidity, *Plasmodium*, Polyparasitism, Splenomegaly

## Abstract

**Background:**

Polyparasitism is still widespread in rural communities of the developing world. However, the epidemiology of polyparasitism and implications for morbidity are poorly understood. We studied patterns of multiple species parasite infection in two rural communities of Côte d’Ivoire, including associations and interactions between infection, clinical indicators and self-reported morbidity.

**Methods:**

Between August and September 2011, two purposely selected rural communities in southern Côte d’Ivoire were screened for helminth, intestinal protozoa and *Plasmodium* infection, using a suite of quality-controlled diagnostic methods. Additionally, participants were examined clinically and we measured haemoglobin level, height, weight and mid-upper arm circumference to determine nutritional status. An anamnestic questionnaire was administered to assess people’s recent history of diseases and symptoms, while a household questionnaire was administered to heads of household to collect socioeconomic data. Multivariate logistic regression models were applied for assessment of possible associations between parasitic (co-)infections and morbidity outcomes.

**Results:**

912/1,095 (83.3%) study participants had complete parasitological data and 852 individuals were considered for in-depth analysis. The rate of polyparasitism was high, with *Plasmodium falciparum* diagnosed as the predominant species, followed by *Schistosoma haematobium*, *Schistosoma mansoni* and hookworm. There were considerable differences in polyparasitic infection profiles among the two settings. Clinical morbidity such as anaemia, splenomegaly and malnutrition was mainly found in young age groups, while in adults, self-reported morbidity dominated. High parasitaemia of *P. falciparum* was significantly associated with several clinical manifestations such as anaemia, splenomegaly and fever, while light-intensity helminth infections seemed to have beneficial effects, particularly for co-infected individuals.

**Conclusions:**

Clinical morbidity is disturbingly high in young age groups in rural communities of Côte d’Ivoire and mainly related to very high *P. falciparum* endemicity. Interactions between helminth infections and *P. falciparum* burden (parasitaemia and clinical morbidity) are evident and must be taken into account to design future interventions.

## Background

Hundreds of millions of people in the developing world are at risk of parasitic diseases, such as malaria and neglected tropical diseases (NTDs) [1-3]. Among the NTDs, parasitic worm (helminth) infections are particularly important in terms of number of people infected and estimated global burden, as expressed in disability-adjusted life years (DALYs) [4-7]. In Côte d’Ivoire, an estimated 33,600 deaths and 2.5 million DALYs were attributable to malaria and NTDs in 2010. These estimates represent 16.6% of the total DALYs and 14.8% of all deaths in Côte d’Ivoire in 2010 [8]. Typical clinical manifestations from infection with *Plasmodium* spp. include anaemia and splenomegaly associated with erythrocyte death and splenic sequestration, respectively [9,10]. Helminth infections (e.g. soil-transmitted helminths, *Schistosoma mansoni* and *Schistosoma haematobium*) are rarely fatal, but cause long-term chronic morbidity [11,12]. This may include anaemia due to blood loss from intestinal or urinary tract bleeding, iron-deficiency linked to nutritional impairment such as malabsorption and other digestive disorders like diarrhoea [13]. Nutritional impairment and competition for nutrients with intestinal parasites further affect the nutritional status leading to malnutrition and impaired child growth [14]. *Schistosoma* spp. infections may cause tissue damage, and hence have been associated with organ pathology mainly driven by migrating parasite eggs in the human body.

To date, most research on parasitic disease-related morbidity focused on single species infections, whilst the health impact due to polyparasitism remains poorly understood [15]. For countries like Côte d’Ivoire where polyparasitism is still widespread [16-18], a deeper mechanistic understanding of multiple species parasite infections is crucial for disease control and the reduction of the burden due to these (co-)infections. Findings from recent studies in different parts of the world are conflicting. For instance, while some studies reported a higher frequency of anaemia in individuals co-infected with *Plasmodium* and helminths, other studies found high anaemia rates in individuals with single species *P. falciparum* infections [19-21]. Intensity of infection plays an important role in shaping morbidity patterns. Ezeamama and colleagues [22] showed strong additive or even multiplicative effects on anaemia in children with high-intensity hookworm and *Schistosoma japonicum* co-infections in the Philippines. In another study carried out in Senegal, light-intensity infections of *S. haematobium* were associated with lower malaria parasitaemia in children, but the opposite was found in Kenyan children where high-intensity infections of both parasites were positively associated [23,24]. Thus, associations and possible inhibitory or favouring mechanisms between different parasite species are of considerable interest, and new research is needed to shed additional light on these issues.

Health effects from multiple species infections are complex due to associations between parasites and possible synergism/antagonism on disease outcome. Additionally, associations are further complicated due to a diversity of proximal and distal risk factors (e.g. socioeconomic status and poor nutrition), as well as demographic, exposure and immunological factors. The aim of the study presented here was to deepen the understanding of the epidemiology of polyparasitism and its implications for morbidity. Residents from two purposely selected communities in Côte d’Ivoire were examined with a suite of diagnostic methods, interviewed with a pre-tested questionnaire and subjected to detailed clinical examinations.

## Methods

### Ethics statement

The study protocol was approved by the institutional research commission of the Swiss Tropical and Public Health Institute (Basel, Switzerland) and received clearance from the ethics committees of Basel (EKBB, reference no. 30/11) and Côte d’Ivoire (reference no. 09-2011/MSHP/CNER-P). District health authorities and village chiefs were informed about the objectives, procedures and potential risks and benefits of the study. Written informed consent was obtained from each individual (and parents/guardians of children aged below 18 years), emphasising that participation is entirely voluntary and that participants can withdraw from the study at any time without further obligation.

At the end of the survey, albendazole (400 mg for participants >2 years and 200 mg for children aged 1-2 years) against soil-transmitted helminthiasis irrespective of infection status was administered. Individuals with a *Schistosoma* infection received praziquantel (40 mg/kg). Participants with clinical malaria (i.e. fever and a positive malaria rapid diagnostic test (RDT)) were given artemisinin-based combination therapy (artesunate-amodiaquine for adults and artemether-lumefantrine for children) and paracetamol against fever. An anti-anaemic treatment in severely anaemic individuals with no malaria symptoms was provided. All treatment regimens were offered free of charge. Data were coded and confidentially treated.

### Study area and population

We purposely selected two rural communities in south and south-central Côte d’Ivoire based on different helminthiases endemicity profiles; Sahoua [25] and Ancien Carrefour [26]. Sahoua borders the Bandama River approximately 160 km north-west of Abidjan and is located in the Taabo health and demographic surveillance system (HDSS) (geographical coordinates: 6°19’20" N latitude, 5°10’30" W longitude) [25]. Ancien Carrefour is a hamlet of Azaguié town in the region of Agnéby-Tiassa, intersected by numerous small rivers and stagnant water bodies and is situated approximately 40 km north of Abidjan (5°37’40" N, 4°01’15" W) [26]. Previous studies in nearby villages revealed high helminth infection prevalences; along the Bandama River mainly *S. haematobium* and hookworm [27-29], and in the Azaguié area mainly *S. mansoni* and soil-transmitted helminth infections [18,26]. Using a cross-sectional epidemiological design, in both villages, all inhabitants (872 in Sahoua and 498 in Ancien Carrefour) were invited to participate, involving parasitological and clinical examinations and questionnaire interviews. The field work was conducted in August and September 2011.

### Field and laboratory procedures

In Sahoua, detailed demographic data (number of household members, names, age, sex and specific identification numbers for each household) were readily available from the Taabo HDSS database. In Ancien Carrefour, a demographic survey was carried out with the assistance of four designated local people to identify all households and to collect demographic information. Additionally, socioeconomic data and behavioural aspects regarding parasitic diseases were gathered at the unit of the household, by administering a pre-tested questionnaire [30]. Finally, a pre-screening among 60 school-aged children was undertaken to clarify the extent of helminth infections. Urine examinations revealed no microhaematuria, which is a useful proxy for *S. haematobium*[31]. Hence, in the subsequent parasitological survey, participants from Ancien Carrefour were asked to provide stool samples exclusively, while participants from Sahoua provided stool and urine samples.

Pre-packed plastic bags containing one stool container (and an additional urine container in Sahoua), labelled with the name and unique identifiers for each participant, were distributed among all households. Household members were asked to return filled containers the next morning. Participants were invited for a finger-prick blood sample that was subjected to an RDT for malaria (ICT ML01 Malaria Pf kit; ICT Diagnostics, Cape Town, South Africa) and thick and thin blood films prepared on microscope slides.

Stool (and urine) specimens and thick and thin blood films were transferred to nearby laboratories. The stool samples were processed as follows. First, a small portion of stool (1-2 g) was fixed in sodium acetate-acetic acid-formalin (SAF) [32]. Second, duplicate Kato-Katz thick smears using 41.7 mg templates [33] were prepared from each stool sample. For the detection of *S. haematobium*, urine samples were subjected to a filtration method [34]. Kato-Katz thick smears and urine filters were examined under a microscope by experienced laboratory technicians. The number of *S. haematobium* (urine filters) and *S. mansoni, Ascaris lumbricoides*, *Trichuris trichiura* and hookworm eggs (Kato-Katz thick smears) were counted and recorded separately. Thick and thin blood films were stained with Giemsa.

For quality control, 10% of the slides were randomly selected and re-examined by a senior microscopist. In case of discordant results, the respective slides were re-examined and the results discussed among the concerned technicians until agreement was found. If results from a specific technician had an error rate above 10%, all slides on that day were re-read.

The SAF-fixed stool specimens and the Giemsa-stained blood films were forwarded to a laboratory in Abidjan. SAF-fixed stool samples were subjected to an ether-concentration technique and the spectrum of intestinal protozoa investigated included *Entamoeba histolytica/E. dispar*, *Entamoeba hartmanni, Entamoeba coli, Endolimax nana, Iodamoeba bütschlii, Giardia intestinalis, Chilomastix mesnili* and *Blastocystis hominis*. Helminth eggs were also recorded for each species separately [35]. Giemsa-stained blood films were assessed for parasitaemia (parasites/μl of blood) and *Plasmodium* species identification under a microscope, following standardised, quality-controlled procedures [36].

Disease-related morbidity was classified into two types; clinical manifestations and self-reported morbidity. Clinical manifestations were assessed during a medical examination by two experienced clinicians. These included liver and spleen enlargement determined by palpation and in case of splenomegaly graded according to the Hackett’s scale [37], anaemia through haemoglobin (Hb) measurement using a HemoCue analyser (HemoCue Hb 301 system; Angelholm, Sweden) and pallor through examination of the inferior conjunctiva. Additionally, body temperature was measured in all participants using an ear thermometer (Braun ThermoScan IRT 4520; Kronberg, Germany) to identify fever cases (≥38.0 °C). Anthropometric measurements, including mid-upper arm circumference (MUAC) (in cm, precision mm), height (in cm) and body weight (in kg, precision 0.5 kg) were taken for subsequent calculation of the nutritional status. Self-reported morbidity was assessed through an anamnestic questionnaire recalling major disease-related symptoms (transient morbidity) such as diarrhoea, abdominal pain, blood in the stool and blood in urine, using a recall period of 2 weeks, and self-reported chronic morbidity (persistent morbidity: discomfort, pain or any other disabling health condition persisting over longer periods).

### Statistical analysis

Data were double-entered and cross-checked in EpiInfo version 3.5.3 (Centers for Disease Control and Prevention; Atlanta, USA). Statistical analysis was performed in Stata version 10.1 (Stata Corp.; College Station, USA). A two-sample analytical approach was employed. The first sample consisted of individuals who had complete parasitological datasets, which was used for basic parasitological and polyparasitism frequency analysis. The second sample included those individuals who, additionally, had complete socioeconomic data and clinical measurements, thus allowed investigating the relationship between co-infection, socioeconomic status and morbidity using multivariate regression models. Two different age cut-offs were used; (i) <5, 5-9, 10-14, 15-24, 25-39 and ≥40 years in sample 1 and (ii) <5, 5-9, 10-18, 19-39 and ≥40 years in sample 2. The rationale to use slightly different age groups was to account for common parasite peak prevalences in sample 1 [16,17] and have suitable cut-offs for the calculation of morbidity indicators (e.g. anaemia and malnutrition) in sample 2.

Classes of intensity for *Schistosoma* spp. and soil-transmitted helminth infections were grouped according to guidelines of the World Health Organization (WHO) [38]. Intestinal protozoa were recorded semi-quantitatively, distinguishing between light (one to five cysts or trophozoites per slide); moderate (one cyst or trophozoite per observation field at a magnification of ×400 or 500); and heavy (more than one cyst or trophozoite per observation field at a magnification of ×400 or 500) [35]. The severity of anaemia was categorised according to WHO guidelines and taking into account for 10 g/l lower cut-offs in African populations [39,40]. The nutritional status for children <5 years and children aged 5-18 years was determined according to available macros for Stata with the new child growth standards and references published by WHO [41]. Indicators for malnutrition in children aged <5 years included wasting (weight-for-height), stunting (height-for-age), underweight (weight-for-age), thinness (body mass index (BMI)-for-age) and MUAC-for-age, while for children aged 5-18 years only stunting, thinness and underweight, the latter only up to the age of 10 years, could be applied as reference measures for nutritional status. BMI and MUAC classes were determined according to Eddleston *et al*. [42] and were used as measures for malnutrition in adults. All nutritional indicators were classified as (i) mild (Z-score <-1 >-2); (ii) moderate (Z-score <-2 >-3); and (iii) severe (Z-score <-3). Splenomegaly was defined as having a palpable spleen of grade 1 or higher according to Hackett’s scale.

A household-based asset approach was used to determine participants’ socioeconomic status, which allowed stratifying individuals into economic groups (wealth quintiles). This approach has been successfully applied and a detailed description is given in a previous study conducted in Côte d’Ivoire [43]. As a measure for the magnitude of inequality between health (here: parasitic infections) and the socioeconomic status, the concentration index (C) was used [44].

For frequency statistics to compare for infection and morbidity rates between different strata, χ^2^ and Fisher’s exact test were applied, as appropriate, while for the mean number of concurrent infections two non-parametric tests (Kruskal-Wallis and the Mann-Whitney test) were used to account for skewed distributions. Comparison for Hb levels by *Plasmodium*-helminth co-infection categories was executed with one-way ANOVA or Kruskal-Wallis test in case of unequal variances.

To estimate associations between parasitic infections and morbidity indicators, multivariate logistic regression models were used. In a first step, bivariate associations were assessed and parasite species and morbidity indicators not showing any significant relationships were removed from further analyses. In each model (poly-) parasitic infection status or infection intensity served as covariates, depending on better fit of the model. A forward stepwise elimination approach was applied for each model, including covariates at a significance level of 0.15. All models were adjusted by age group, sex and socioeconomic status. Significant relationships between an outcome (infection with a parasite or a specific morbidity indicator) and explanatory variables (infection status or intensity of infection) were expressed as adjusted odds ratios (ORs) with corresponding 95% confidence intervals (CIs). Regression analysis for clinical outcomes was performed for children/adolescents aged <18 years and adults aged ≥18 years separately, accounting for age-specific morbidity and parasitic infection patterns.

Co-infection and co-morbidity pairs of the multivariate regression models for children/adolescents comprised (i) *P. falciparum*-malnutrition comorbidity; (ii) *P. falciparum*-hookworm co-infection; and (iii) *P. falciparum*-*S. haematobium* co-infection. Considering the high prevalence of *P. falciparum* in this age range and that morbidity depends on parasitaemia, *P. falciparum* infections with a parasitaemia of >500 parasites/μl of blood were used instead, while malnutrition only included moderate and severe cases. For the adult models, co-infection with *P. falciparum*-*S. mansoni* was of main interest as well as hookworm-*S. mansoni* co-infection. All significant associations were presented for the four co-infection categories: (i) none of the two conditions; (ii) mono-infection species 1; (iii) mono-infection species 2; and (iv) co-infection with both species and were expressed as OR with 95% CI.

## Results

### Study participation and operational results

**Figure 1:**
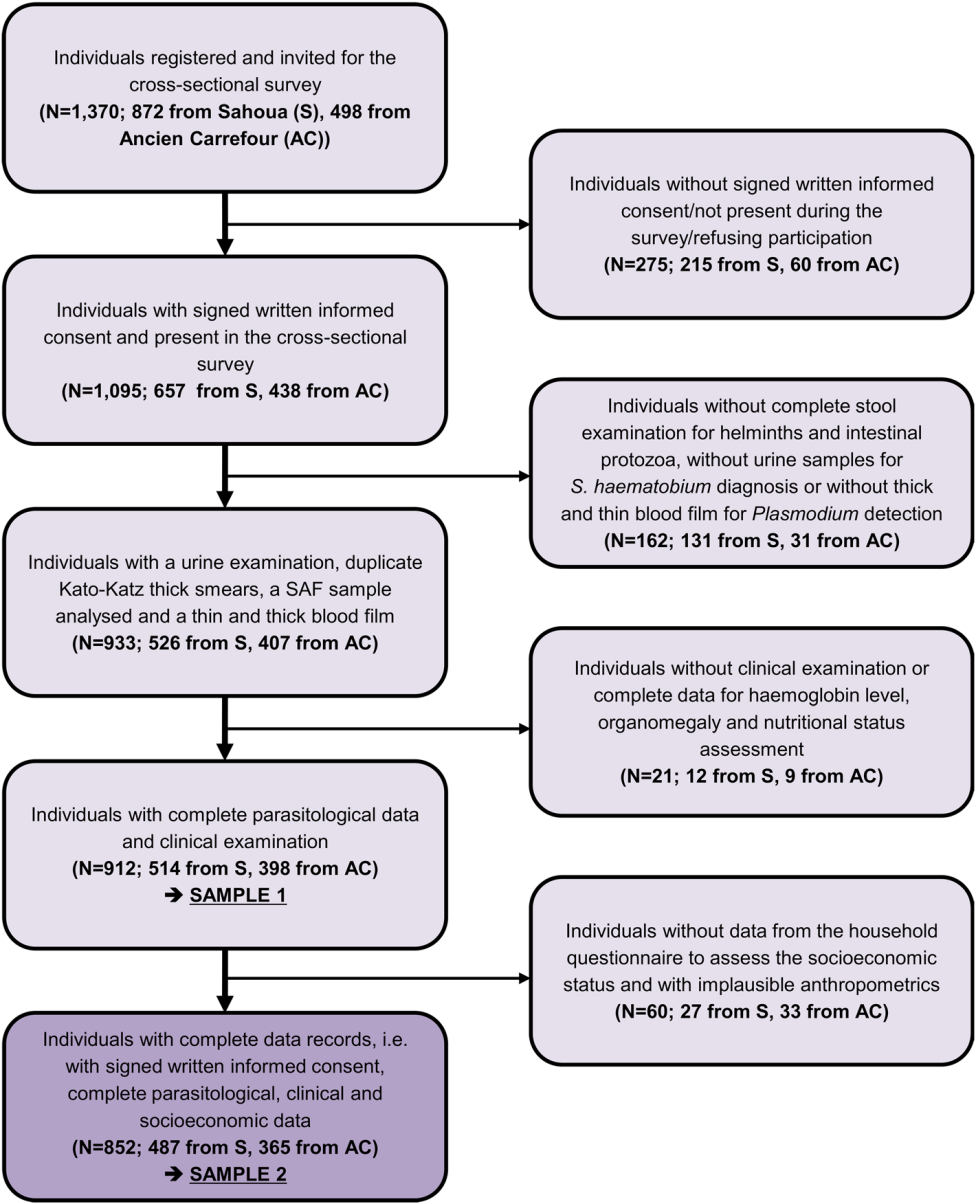
**Flow chart illustrating study participation and compliance.** The cross-sectional surveys were conducted in two rural settings in south and south-central Côte d’Ivoire in August and September 2011. Urine examination was done in Sahoua exclusively. AC = Ancien Carrefour, S = Sahoua.

Overall, 1,095 out of a total of 1,370 inhabitants in the two communities participated in the survey, resulting in an overall compliance of 79.9%. Considering inter-village differences, the study participation was higher in Ancien Carrefour (88.0%) compared to Sahoua (75.3%). Figure 1 gives a flow chart, showing the compliance to different stages of the study. For 912 individuals, complete parasitological data and full records from the clinical examination were available. These data were summarised as sample 1 and used for the analysis of parasite prevalence profiles and polyparasitism. Sixty individuals were excluded either for missing information on socioeconomic status or for implausible anthropometric measurements, resulting in 852 records assigned to sample 2, which was utilised for further analyses on parasite associations and relationships with morbidity indicators. Female and male participants were equally distributed in both samples used: 463 females and 449 males in sample 1, while sample 2 consisted of 431 female and 421 male participants. The distribution among males and females within the age groups showed no significant difference in both samples (sample 1: χ^2^ = 6.79, degrees of freedom (d.f.) = 5, p = 0.236; sample 2: χ^2^ = 4.50, d.f. = 4, p = 0.343).

### Frequencies of parasitic infections and comparison for sociodemographic variables

The *S. haematobium* prevalence in Sahoua was 25.9%. *S. mansoni* was much more prevalent in Ancien Carrefour than in Sahoua (28.4% *vs.* 1.9%, p <0.001). Concerning infection rates with soil-transmitted helminths, similar patterns were found in both communities; *A. lumbricoides* and *T. trichiura* infections were rarely found, hookworm being the predominant species with prevalences of 32.4% and 26.8% in Ancien Carrefour and Sahoua, respectively. Most of the soil-transmitted helminth infections were of light intensity. The endemicity profiles of the two settings differed not only for *Schistosoma*, but also for intestinal protozoa infections. In Sahoua a higher affection by intestinal protozoa was found in terms of prevalence and intensity of infection. Overall, the three most common intestinal protozoa species were *E. coli*, *B. hominis* and *E. nana*, with prevalences of 33.2%, 28.7% and 22.3%, respectively. The known pathogenic protozoan species *G. intestinalis* and *E. histolytica/E. dispar* were detected in 105 (11.5%) and 86 (9.4%) participants, respectively. In both localities three different *Plasmodium* species were identified; *P. falciparum* was the predominant species (overall prevalence in Ancien Carrefour and Sahoua was 70.1% and 59.7%, respectively), whilst *P. malariae* and *P. ovale* were detected in 33 (3.6%) and 3 (0.3%) of the participants, respectively.

Males were significantly more often infected with hookworm (males: 34.7%, females: 24.0%, p <0.001) and *S. mansoni* (males: 15.8%, females: 11.2%, p = 0.043) than females.

Several parasites showed significant associations with age, such as both *Schistosoma* species (*S. haematobium*: χ^2^ = 93.15, d.f. = 5, p <0.001; *S. mansoni*: χ^2^ = 51.49, d.f. = 5, p <0.001), hookworm (χ^2^ = 61.07, d.f. = 5, p <0.001) and three intestinal protozoan species (*E. coli*, *E. nana* and *G. intestinalis*). *S. haematobium*, *S. mansoni* and hookworm infections showed peak prevalences in the age groups of 10-14 years (prevalence: 35.0%), 15-24 years (prevalence: 24.0%) and 25-39 years (prevalence: 39.7%), respectively. The pathogenic intestinal protozoon species *G. intestinalis* was more often found in young children (peak in the age group of 5-9 years with 22% infected), while the prevalence of non-pathogenic intestinal protozoa increased with age (peak in individuals aged 25-39 years). Malaria parasites were significantly more often found in younger individuals, and the three individuals identified to harbour *P. ovale* all belonged to the youngest age group of under-fives.

Hookworm and *S. mansoni* were significantly associated with a lower socioeconomic status and more prevalent in participants from poorer households (concentration indices and standard errors: C = -0.0624, SE = 0.0306 and C = -0.2126, SE = 0.0427, respectively).

### Polyparasitism

**Figure 2:**
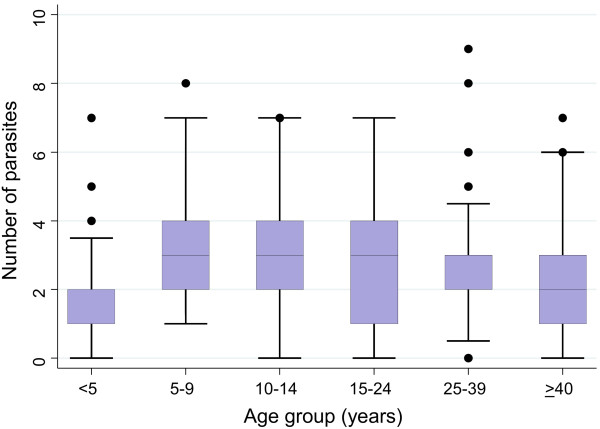
**Number of concurrent parasitic infections, stratified by age group.** Rate of polyparasitism among 912 study participants with complete parasitological data. Box plot: boxes illustrate the 25^th^ and 75^th^ percentiles (ptile), while the whiskers indicate the adjacent lower and upper values (most extreme values which are within 25^th^ ptile - 1.5*(75^th^-25^th^ ptile) and 75^th^ ptile + 1.5*(75^th^-25^th^ ptile), respectively). The median is shown by the line within the boxes, outliers are indicated with dots.

Polyparasitism was common; on average, a study participant harboured 2.5 concurrent parasitic infections. The maximum number of parasite species found in the same host was nine. The median number of infections significantly differed between age groups (Kruskal-Wallis, p <0.001) illustrated in Figure 2, showing a peak in the age groups of 5-9 years (median: 3 parasites/individual, range: 1-8 parasites) and 10-14 years (median: 3 parasites/individual, range: 0-7 parasites), hence school-aged children showed the highest extent of polyparasitism. Females were slightly less affected by multiple species parasite infections compared to their male counterparts (Wilcoxon rank-sum with p = 0.020). Considering the high endemicity of *P. falciparum* (overall prevalence: 64.3%), the major co-infections identified were concurrent infections with *P. falciparum* and helminths, particularly hookworm (18.1%), *S. haematobium* (10.0%) and *S. mansoni* (8.9%). Co-infection patterns differed with age group. In younger age groups, where infections with helminths were less common, concurrent infections with *P. falciparum* and pathogenic intestinal protozoa like *G. intestinalis* and *E. histolytica/E. dispar* added up to the polyparasitic burden with a peak prevalence of 19.2% and 9.0%, respectively, in children aged 5-9 years.

### Paired associations between parasite species

The findings from the multivariate regression analysis revealed a significant positive association in both ways for *P. falciparum* and *S. mansoni* with adjusted ORs of 2.03 and 2.14, respectively. All 78 (66.7%) *S. mansoni*-positive individuals who were co-infected with *P. falciparum*, exclusively had low *P. falciparum* parasitaemia with <500 parasites/μl blood (data not shown). *S. mansoni* infection shared a strong positive association with hookworm infection (OR = 2.78, p <0.001) and *vice versa* (OR = 2.78, p < 0.001). Most intestinal protozoa species showed significant positive associations between each other. *E. coli*, for example, was associated with *E. histolytica/E. dispar* (OR = 3.19), *E. nana* (OR = 4.68) and *I. bütschlii* (OR = 5.93) (all p <0.001). Significant associations from multivariate regression models between a particular parasite species and any other parasite, sex, age group and socioeconomic status are presented in Additional file 1.

### Clinical and self-reported morbidity

**Figure 3:**
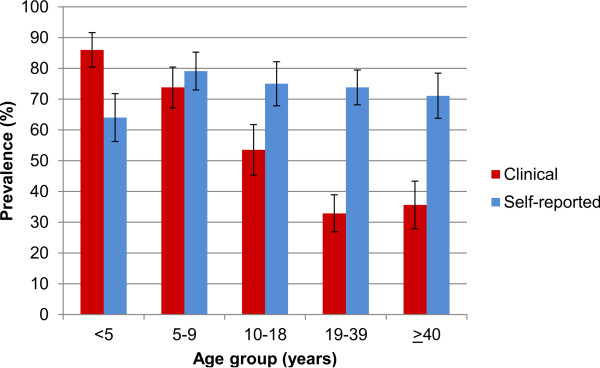
**Prevalence of at least one clinical and/or self-reported morbid sequelae, stratified by age group.** Clinical outcomes included: anaemia, splenomegaly, pallor, fever and malnutrition (z-scores <-2), while self-reported morbidity comprised reported symptoms of diarrhoea, abdominal pain, blood in the stool, blood in urine and chronic morbidity. All morbidity data were assessed during medical examination in 852 study participants.

**Figure 4:**
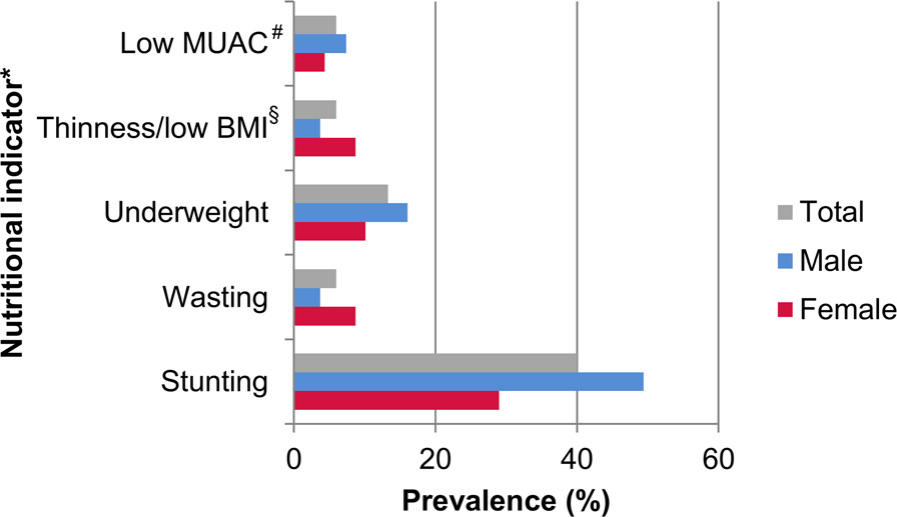
**Prevalence of different nutritional indicators used to assess malnutrition in children aged <5 years, stratified by sex.** *Considered are only individuals showing moderate or severe signs for malnutrition; ^#^MUAC = mid-upper-arm-circumference; ^§^BMI = body mass index (weight / height^2^).

The assessment for clinical morbidity revealed the prevalence for hepatomegaly, splenomegaly and anaemia of 0.2%, 15.7% and 19.5%, respectively among 852 individuals from both communities. Figure 3 depicts the extent of clinical manifestations assessed and self-reported symptoms and recent histories of disease reported during clinical examination, stratified by age group. Clinical morbidity mainly affected young age groups, while self-reported morbidity was common among all age groups. Besides age, morbidity patterns also differed by sex. In the most affected group of children under 10 years of age, splenomegaly and malnutrition were significantly more often found in boys than in girls (38.8% *vs.* 28.0% and 42.4% *vs.* 28.7%, respectively). Anaemia did not differ between boys and girls, but was associated with female sex in adulthood (age >18 years). 19% of all study participants showed any sign of moderate or severe nature for malnutrition (for details see Additional file 2). Stunting with a prevalence of 40% was by far the most common sign for malnutrition in the youngest and most affected age group. Additionally, stunting was significantly more often found in males than in females (49.4% *vs.* 29.0%) (Figure 4).

### Associations between parasitic infections and morbidity

Table 1 highlights all significant associations of clinically assessed and self-reported morbidity with any parasite infection status or intensity derived from the multivariate regression analysis. Anaemia, splenomegaly and fever all showed a strong positive association with *P. falciparum* parasitaemia. Splenomegaly was significantly associated with *P. malariae*, which was predominantly found in young age groups.

**Table 1:** Statistically significant associations between parasitic infections and clinically assessed or self-reported morbidity from multivariate regression analysis

**Morbidity**^**§**^	**Association**	**Adjusted OR (95% CI)**	**p-value**
**Clinically assessed morbidity**			
Anaemia^*2+,7i,8i,9i,10i,12i,14*^	*P. falciparum* parasitaemia (>5,000 parasites/μl of blood)	3.54 (1.63, 7.66)	0.001
	*E. histolytica/E. dispar* intensity (++)	4.43 (1.79, 10.97)	0.001
	*S. mansoni* intensity (+)	0.29 (0.10, 0.86)	0.025
	Hookworm intensity (+)	0.60 (0.37, 0.99)	0.045
Splenomegaly^*3+,6+,7i,9i,10i,14*^	*P. falciparum* parasitaemia (>5,000 parasites/μl of blood)	6.26 (2.60, 15.03)	<0.001
	*P. falciparum* parasitaemia (501-5,000 parasites/μl of blood)	3.71 (1.80, 7.64)	<0.001
	*P. malariae*	2.28 (1.01, 5.18)	0.048
Malnutrition (z-score <-2)^*9+,13,14,15*^	*S. mansoni* intensity (+)	0.32 (0.11, 0.93)	0.036
	*S. haematobium*	0.55 (0.31, 0.98)	0.041
Pallor^*7i,11i,14*^	*S. mansoni*	0.40 (0.23, 0.70)	0.001
	*E. histolytica/E. dispar*	0.52 (0.29, 0.93)	0.026
Fever (≥38.0 °C)^*3+,6+,14*^	*P. falciparum* parasitaemia (>5,000 parasites/μl of blood)	4.19 (1.35, 13.03)	0.013
**Self-reported morbidity**			
Diarrhoea^*6i,9+,11i,12i,14,16*^	*S. mansoni* intensity (++)	3.33 (1.35, 8.23)	0.009
Abdominal pain^*9i,14,15*^	*S. mansoni*	2.54 (1.66, 3.89)	<0.001
	*G. intestinalis* intensity (++)	2.14 (1.17, 3.93)	0.013
Blood in the stool^*7+,9+,10i,12i,13,14*^	*S. haematobium*	2.20 (1.34, 3.62)	0.002
	*S. mansoni*	2.13 (1.27, 3.57)	0.004
	*P. falciparum*	0.58 (0.38, 0.89)	0.013
Blood in urine^*7i,8i,9i,11i,12i,13,14*^	*S. haematobium* intensity (+)	2.77 (1.61, 4.77)	<0.001
	*S. haematobium* intensity (+++)	11.08 (4.75, 25.83)	<0.001
	*S. mansoni*	0.29 (0.12, 0.72)	0.008
Chronic morbidity^*6i,7i,8i,9i,11i,12i,14,15*^	No. of concurrent pathogenic infections (≥3 parasites)	0.43 (0.21, 0.88)	0.022
	*S. haematobium*	2.25 (1.35, 3.75)	0.002
	*S. mansoni*	0.38 (0.19, 0.74)	0.004

Reference categories: parasite infection status or intensity: no infection. Helminth infection intensities: (+) = light, (++) = moderate, (+++) = heavy.

^§^Covariates further included in each model for adjustment (+ = infection with, i = intensity of infection): 1 = *P. falciparum*, 2 = *P. malariae*, 3 = *S. haematobium*, 4 = *S. mansoni*, 5 = Hookworm, 6 = *E. histolytica/E. dispar*, 7 = *E. coli*, 8 = *E. nana*, 9 = *I. bütschlii*, 10 = *G. intestinalis*, 11 = *C. mesnili*, 12 = *B. hominis*, 13 = sex, 14 = age group, 15 = socioeconomic status, 16 = number of concurrent pathogenic infections (0, 1, 2, and 3 or more).

**Figure 5:**
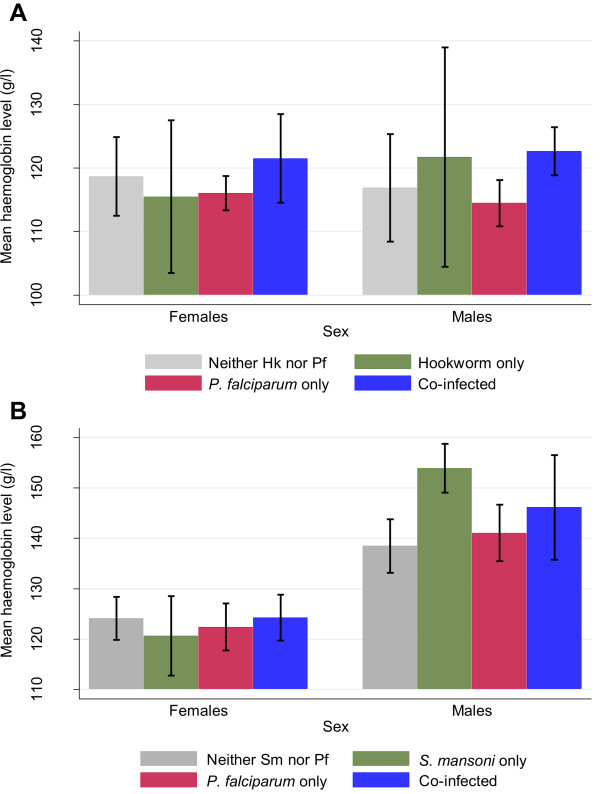
**Comparison of mean haemoglobin levels in different age groups, stratified by sex in relation to helminth-*P. falciparum *co-infection from one-way ANOVA and Kruskal-Wallis test. A**: Comparison of mean haemoglobin (Hb) level in children aged 5-11 years (n = 224) shown for both sexes with different infection categories for hookworm (Hk) and *P. falciparum* (Pf). One-way ANOVA analysis showed significant differences in Hb for the total sample (not shown, p = 0.008) and in males (p = 0.028) **B**: Comparison of mean Hb level in participants aged 16-39 years (n = 243) shown for both sexes with different infection categories for *S. mansoni* (Sm) and *P. falciparum* (Pf). Kruskal-Wallis test showed significant differences in Hb for the total sample (not shown, p = 0.021) and in males (p = 0.015). Pregnant women (n = 31) were excluded from the analysis since pregnancy significantly lowers the Hb levels. In this sample of women aged 16-39 years, pregnant women had a mean Hb of 113.5 g/l *vs.* 123.3 g/l in non-pregnant women (p = 0.001).

Individuals presenting light-intensity infection with *S. mansoni* and hookworm had significantly lower ORs for anaemia. Effects on Hb levels due to infection with these helminths and co-infection with *P. falciparum* are illustrated in Figure 5. Children aged 5-11 years overall had significantly higher Hb levels if co-infected with hookworm (mean: 122.3 g/l, 95% CI: 119.0, 125.5 g/l) compared to individuals with *P. falciparum* mono-infection (mean: 115.4 g/l, 95% CI: 113.2, 117.5 g/l). In adolescents and adults aged 16-39 years, individuals with *S. mansoni* mono-infection were found to have significantly higher Hb values (mean: 143.7 g/l, 95% CI: 136.3, 151.1 g/l) compared to individuals with neither infection (mean: 131.2 g/l, 95% CI: 127.6, 134.8 g/l) or with *P. falciparum* mono-infection (mean: 132.1 g/l, 95% CI: 127.9, 136.2 g/l). This effect from helminth infections on Hb was found in male individuals only. Malnutrition was negatively associated with both *Schistosoma* species. The association between pallor and *S. mansoni* confirmed the negative direction also observed in the association between anaemia and light-intensity infections of this helminth species.

Although *S. mansoni* (particularly light-intensity infections) seemed to have beneficial effects on clinical outcomes like anaemia and malnutrition, a significant positive association was found with several symptoms involving the digestive tract such as diarrhoea, abdominal pain and blood in the stool (all p <0.05). Self-reported blood in urine and blood in the stool were both positively associated with *S. haematobium* infection. Eggs of *S. haematobium* are expected to be excreted in urine, but five out of the 126 (4%) individuals identified with *S. haematobium* were found to excrete eggs also in the stool.

Self-reported chronic morbidity was negatively associated with the highest category of concurrent infections with pathogenic parasites (non-pathogenic intestinal protozoa excluded), harbouring three or more pathogenic species (p = 0.022), but the association was of positive direction for *S. haematobium*-infected individuals (p = 0.002).

Table 2 shows the key findings from the multivariate regression analysis for the relationship between co-infection and selected morbidities in children/adolescents and adults. An infection with *P. falciparum* (>500 parasites/μl of blood) in combination with malnutrition added up to the risk of having clinical manifestations such as anaemia and splenomegaly in children <18 years of age. The relationship of *P. falciparum* (>500 parasites/μl of blood)*-S. haematobium* co-infection was not of an additive nature but all three infection categories showed significantly higher ORs for splenomegaly among children.

**Table 2:** Associations between specific morbidities and co-infection/co-morbidities in children/adolescents aged <18 years and adults ≥18 years from multivariate regression analysis

**Morbidity**^**§**^	**N**	**Co-infection/co-morbidity category**	**Adjusted OR (95% CI)**	**p-value**
**Children/adolescents aged <18 years (n = 466)**				
Anaemia^3i,4i,6i,14,16^	188	No hookworm/no ${\text{P. falciparum}}_{500+}^{\Delta }$	1.00 (reference)	
	170	*P. falciparum*_500+_ only	1.90 (1.15, 3.14)	0.012*
	66	Hookworm only	0.29 (0.09, 0.88)	0.030*
	42	Co-infected	0.84 (0.34, 2.05)	0.699
Anaemia^3i,4i,5+,6i,14^	181	Not malnourished/no *P. falciparum*_500+_	1.00 (reference)	
	141	*P. falciparum*_500+_ only	1.94 (1.05, 3.57)	0.034*
	73	Malnourished only	2.21 (1.11, 4.41)	0.024*
	71	Malnourished/*P. falciparum*_500+_	4.62 (2.32, 9.21)	<0.001*
Splenomegaly^2+,6i,13,14^	191	No *S. haematobium*/no *P. falciparum*_500+_	1.00 (reference)	
	174	*P. falciparum*_500+_ only	3.10 (1.80, 5.31)	<0.001*
	63	*S. haematobium* only	2.47 (1.11, 5.49)	0.026*
	38	Co-infected	3.48 (1.49, 8.15)	0.004*
Splenomegaly^2+,3+,6i,13,14^	181	Not malnourished/no *P. falciparum*_500+_	1.00 (reference)	
	141	*P. falciparum*_500+_ only	1.91 (1.08, 3.40)	0.027*
	73	Malnourished only	0.70 (0.32, 1.53)	0.374
	71	Malnourished/*P. falciparum*_500+_	3.26 (1.66, 6.40)	0.001*
**Adults aged ≥18 years (n = 384)**				
Anaemia^6i,8+,13^	186	No *S. mansoni*/no *P. falciparum*	1.00 (reference)	
	121	*P. falciparum* only	1.03 (0.52, 2.03)	0.940
	34	*S. mansoni* only	0.47 (0.10, 2.15)	0.332
	43	Co-infected	0.13 (0.02, 0.99)	0.049*
Pallor^8i,12i,13,15^	186	No *S. mansoni*/no *P. falciparum*	1.00 (reference)	
	121	*P. falciparum* only	0.81 (0.47, 1.41)	0.458
	34	*S. mansoni* only	0.74 (0.28, 2.01)	0.560
	43	Co-infected	0.23 (0.07, 0.71)	0.011*
Chronic morbidity^5+,9i,11i,13,14,15^	186	No *S. mansoni*/no *P. falciparum*	1.00 (reference)	
	121	*P. falciparum* only	0.61 (0.37, 1.01)	0.052
	34	*S. mansoni* only	0.53 (0.22, 1.31)	0.168
	43	Co-infected	0.16 (0.06, 0.46)	0.001*
Abdominal pain^14,15^	186	No *S. mansoni*/no *P. falciparum*	1.00 (reference)	
	121	*P. falciparum* only	0.63 (0.36, 1.10)	0.105
	34	*S. mansoni* only	1.74 (0.80, 3.78)	0.163
	43	Co-infected	2.79 (1.38, 5.64)	0.004*
Abdominal pain^14,15^	206	No *S. mansoni*/no hookworm	1.00 (reference)	
	34	*S. mansoni* only	2.15 (0.99, 4.64)	0.051
	101	Hookworm only	1.02 (0.59, 1.78)	0.946
	43	Co-infected	3.27 (1.61, 6.63)	0.001*

*Statistically significant with a p-value <0.05.

^Δ^*P. falciparum* parasitaemia of >500 parasites/μl of blood.

^§^Not significant covariates further included in each model for adjustment (+ = infection with, i = intensity of infection): 1 = *P. falciparum*, 2 = *P. malariae*, 3 = *S. haematobium*, 4 *= S. mansoni*, 5 *=* hookworm, 6 *= E. histolytica/E. dispar*, 7 *= E. coli*, 8 *= E. nana*, 9 *= I. bütschlii*, 10 *= G. intestinalis*, 11 *= C. mesnili*, 12 *= B. hominis*, 13 = sex, 14 = age group, 15 = socioeconomic status, 16 = malnutrition (moderate or severe).

The pattern of negative associations between helminth infections and several morbidity outcomes found in the single-species models were confirmed in the co-infection models. Children infected with hookworm only had significantly lower ORs for anaemia. Additionally, hookworm had an antagonistic effect in relation to *P. falciparum* infection since co-infection with hookworm and a *P. falciparum* parasitaemia >500 parasites/μl of blood was not associated with anaemia. In adults, an antagonistic relationship was found for *P. falciparum*-*S. mansoni* co-infections; individuals co-infected had significantly lower ORs for several morbid sequelae such as anaemia, pallor and recent history of chronic disease.

*S. mansoni* infection among adults, on the other hand, was found to be significantly positively associated with self-reported abdominal pain in combination with other parasites, such as *P. falciparum* and hookworm.

## Discussion and conclusions

Since 2010, the population of Sahoua, located at the northern edge of the Taabo HDSS in south-central Côte d’Ivoire, has benefitted from multiple rounds of deworming, targeting schistosomiasis, soil-transmitted helminthiasis and lymphatic filariasis, which are all endemic in this zone [28,45]. However, the prevalence of hookworm infection was still considerable (26.8%), but most of the infections were of light intensity. One quarter of all *S. haematobium* infections were of high intensity (≥50 eggs/10 ml of urine). The second study location, Ancien Carrefour, has not been subjected to regular deworming before. We found hookworm and *S. mansoni* prevalences of 32.4% and 28.4%, respectively, indicating a moderate-risk community [46]. *Plasmodium* infection was common in both settings. Consequently, polyparasitism was prevalent, even though varying in parasite species composition, which is in line with earlier studies conducted in rural areas of Côte d’Ivoire [16-18]. In fact, less than 10% of the study participants who had complete parasitological data were free of any of the parasites investigated. It should be noted that the true dimension of multiple-species infections is likely to be higher, considering that only single stool, urine and finger-prick blood samples were taken and analysed microscopically. It is widely acknowledged that egg-output of *S. mansoni* shows important intra-stool and day-to-day variation, and hence multiple Kato-Katz thick smears are necessary to increase diagnostic sensitivity [18,47-49]. Despite the likely underestimation of the true prevalence of single and multiple species parasitic infections, we consider the data as meaningful to reveal implications on disease-related morbidity taking into account that those infections missed were most likely of light intensity, whereas disease burden is a consequence of infection intensity [50,51]. The extent of polyparasitism and species combinations showed significant variation by age group. School-aged children were found to have the highest number of concurrent infections, while preschool-aged children less often showed co-infection with three parasites or more (11.2%). The youngest individuals were mainly affected by *Plasmodium* and intestinal protozoa. Helminth infections were most common in the school-aged and adult populations. Peak prevalences varied from species to species; for instance children aged 10-14 years for *S. haematobium*, adolescents and young adults for *S. mansoni* and adults for hookworm. These findings are consistent with previous observations [16,17,52].

Several significant associations between parasite species are worth highlighting; some of which have been discussed before, particularly the co-infection of hookworm and *S. mansoni*[53-55]. It is conceivable that this is strongly related to shared risk factors, such as poor sanitation and hygiene behaviour [26,43,56]. The same conclusions might be drawn for the various associations found between different intestinal protozoa species. Transmission occurs mainly through the faecal-oral pathway, thus by ingestion of contaminated food and water [57]. This issue might explain why we could detect several intestinal protozoa species already in early childhood compared to helminth infections that were more prevalent in older age groups. Infants are restricted in their movement and are less exposed to schistosome-infested water bodies or open defecation grounds. Tackling poor hygiene and improve access to clean water and sanitation offers the opportunity to fight these infectious diseases. New approaches, such as community-led total sanitation (CLTS) exist and a pilot project conducted in the Taabo HDSS showed promising results [43].

As parasite species varied for different age groups, so did clinical manifestations (i.e. anaemia and splenomegaly), which mainly occurred in children. In adults, morbidity patterns were mainly driven by chronic, yet subtle but still disabling morbidities, as assessed by self-reported symptoms and recent history of chronic disease. The transition from acute and clinical morbidity to more chronic conditions in older age is explained by the constant exposure to these parasites in highly endemic areas, and the slowly acquired protective immunity [58,59]. Our findings confirm that the burden of disease increases with parasite load. Interestingly, *S. mansoni* infection seemed to have a beneficial effect on anaemia in case of light-intensity infection, but was at the same time associated with a number of gastro-intestinal symptoms with higher intensity. Furthermore, anaemia was significantly associated with high-intensity infections of *P. falciparum*, *E. histolytica/E. dispar* in all individuals and *S. haematobium* in children aged <9 years (data not shown). These results are in line with findings from earlier studies on disease outcome in relation to *P. falciparum* parasitaemia and intensity of *S. haematobium*[50,51] and justify one of the defined primary goals of control programmes for high endemicity areas, namely to reduce morbidity by periodic deworming, and thus eliminate moderate and high infection intensities but not necessarily cure all infections. Malnutrition could not directly be associated with any parasitic infection and indicates the need to integrate information on local dietary habits for future surveys on assessing this health consequence. In our study, malnutrition nevertheless served as an additional condition that was shown to be associated with other disease outcomes, as it is the case for anaemia and splenomegaly in children. Malaria remains the most important parasitic disease in terms of prevalence and clinical outcomes, high parasitaemia being associated with splenomegaly and fever besides anaemia. Long-lasting insecticidal nets (LLINs) as preventive measures are promoted in health centres and efforts have been made to increase coverage [60,61]. Two out of three of the surveyed households possessed a LLIN, but coverage differed between study sites with 90% in Ancien Carrefour and 43% in Sahoua. Despite a high coverage in Ancien Carrefour, the malaria parasite rate, and consequently the disease-related burden, was striking. In recent years, bed net coverage has increased considerably in Côte d’Ivoire [61,62], but still needs further scaling up actions [61,63]. Additionally, local knowledge, attitudes and practices towards malaria and other parasitic diseases must be addressed, as they influence the use of preventive measures, help-seeking and treatment behaviour, and risk-related behaviour [30,60].

Another interesting finding of this study is the indication of protective effects on anaemia in individuals co-infected with helminths (i.e. hookworm and *S. mansoni*). This confirms earlier studies carried out in comparable rural settings elsewhere in West Africa [20,23]. The underlying mechanisms of such a protective effect need further scientific inquiry, but might be explained by the immunomodulatory effects that helminths are known for [64]. In Senegalese school-aged children, chronic schistosomiasis influenced the humoral immune response against malaria antigens by specifically increasing IgG1 and IgG3 Abs levels, which are thought to play a role in protection during human malaria [65]. Moreover, one has to keep in mind that most helminth infections detected were of light intensity, a different relationship may be observed if worm loads are higher. In general, our findings suggest that a co-infection does not automatically mean more disease-related pathology. Individuals who were diagnosed with three or more concurrent pathogenic infections were found to be negatively associated with a recent history of chronic disease. Co-infecting parasites are competing for the limited resources within a single host, thus some combinations of parasite species may reduce disease burden due to inhibition of growth of the other one, especially if the inhibited species causes more pronounced morbidity. In our study we present mainly the inhibitory effects of interactions between parasite species, while in other studies co-infection was related with a higher risk of disease-related morbidity (i.e. anaemia) [21]. In a next step, we will further investigate the directions of interactions between species and its implications for morbidity and additionally investigate their magnitude. Interaction measures like the synergistic index, as defined by Rothman [66] and initially used in clinical case-control studies, offer new ways to assess the magnitude and direction of additive interaction due to co-infection. Ezeamama and colleagues [22], for example, showed that moderate- to heavy-intensity infections of *S. japonicum* and *T. trichiura* were associated with higher odds of anaemia with a synergy index (SI) of 2.9. As a consequence of existing interactions between parasites, treatment campaigns against specific diseases should be conducted with caution and should consider local patterns of co-infection with other species since treating one parasite may exacerbate the consequences to another one. Furthermore, co-infection may influence the effect and efficacy of drugs or vaccines [67]. Specific drugs and agents have been shown to have an impact on different kinds of parasites, like artemisinin-based combination therapy, which is used to treat malaria but also has an effect on *Schistosoma* infection [68,69] or ivermectin that is used in the global programme for eliminating lymphatic filariasis, and also impacts on soil-transmitted helminthiasis [70]. Thus considering the co-occurrence of different parasitic species should also influence the choice of treatment to be applied.

We conclude that multiple-species parasite infections are common in rural parts of Côte d’Ivoire, explained by social-ecological contexts that foster the presence and transmission of these diseases, but that there is small-scale heterogeneity. Taken together, our findings and other recent studies on polyparasitism imply the need for adaptation of future interventions towards integrated control. Treatment campaigns will serve as the backbone of interventions, but must be combined with other control interventions to reduce parasite intensity and thus morbidity [71]. Furthermore, the interactions between parasites are evident and may have implications on morbidity, such as anaemia. Treatment plans should therefore be adapted to local co-infection risk profiles in terms of combined treatment of several infections to avoid exacerbation of one disease by treating the other and in terms of most appropriate drugs to profit of substances active against a range of different species. The fact that infectious diseases like malaria, schistosomiasis, soil-transmitted helminthiasis and intestinal protozoa infections are mainly driven by social-ecological systems [43,49,60,72,73] provides an opportunity to fight them as a whole by addressing these factors. It goes without saying that issues of underdeveloped infrastructure and water and sanitation provision in rural areas of Côte d’Ivoire cannot be improved from one day to another, but new promising approaches do exist. Interventions like CLTS incorporate whole communities and place emphasis on hygiene education taking into account local knowledge, attitudes and practices and schedule concrete action plans determined by the community themselves with the overall goal to achieve open defecation-free status in their village [43]. Programmes like this target on higher acceptance and involvement of the local population and present new ways of more integrated control of parasitic infections that may serve as an example to be adapted.

## Abbreviations

Abs: Antibodies; BMI: Body mass index; C: Concentration index; CI: Confidence interval; CLTS: Community-led total sanitation; DALY: Disability-adjusted life year; Hb: Haemoglobin; HDSS: Health and demographic surveillance system; LLIN: Long-lasting insecticidal net; MUAC: Mid-upper arm circumference; NTD: Neglected tropical disease; OR: Odds ratio; RDT: Rapid diagnostic test; SAF: Sodium acetate-acetic acid-formalin; SI: Synergy index; WHO: World Health Organization.

## Competing interests

The authors declare that they have no competing interests.

## Authors’ contributions

EH, EKN, JU and GR designed the study; EH, RBY, CAH, TS, BAK, KDS, MO, EKN and GR implemented the study; EH, RBY, CAH, TS and BAK managed the data; EH analysed the data and wrote the first draft of the paper; TS, JU and GR contributed to data analysis and helped interpreting the results; RBY, BAK, TS, JU and GR revised the manuscript and provided important intellectual content. All authors read and approved the final version of the manuscript before submission.

## Supplementary Material

Additional file 1 **Statistically significant associations between parasites species from multivariate regression analysis.** This file can be viewed with: Adobe Acrobat reader (url: http://get.adobe.com/uk/reader/). Click here for file

Additional file 2 **Prevalence of clinical morbidity and indicators for malnutrition by sex and age group.** This file can be viewed with: Adobe Acrobat reader (url: http://get.adobe.com/uk/reader/). Click here for file
