# Supplementary material for: The epidemiology of polyparasitism and implications for morbidity in two rural communities of Côte d’Ivoire
Source: Parasit Vectors. 2014 Feb 25;7:81. doi: 10.1186/1756-3305-7-81 (PMC3942297; doi:10.1186/1756-3305-7-81)
Supplement: Additional file 1 — Statistically significant associations between parasites species from multivariate regression analysis. This file can be viewed with: Adobe Acrobat reader (url: http://get.adobe.com/uk/reader/). [file 1756-3305-7-81-S1.pdf]

Additional file 1. Statistically significant associations between parasites species from multivariate regression analysis

Reference categories: sex: female, age group: 0-4 years, and wealth quintile: most poor

Parasite	Association	Adjusted OR (95% CI)
Plasmodium		
P. falciparum[8,12]	Age group (10-18 years)	0.28 (0.16, 0.51)
	Age group (19-39 years)	0.11 (0.06, 0.19)
	Age group (≥40 years)	0.07 (0.04, 0.13)
	S. mansoni	2.03 (1.29, 3.19)
P. malariae[1,5,10]	Age group (10-18 years)	0.08 (0.01, 0.67)
	Age group (19-39 years)	0.13 (0.03, 0.63)
	Age group (≥40 years)	0.11 (0.01, 0.93)
Schistosoma		
S. haematobium[13]	Age group (5-9 years)	6.32 (2.96, 13.51)
	Age group (10-18 years)	9.76 (4.53, 21.04)
	S. mansoni	0.11 (0.03, 0.34)
S. mansoni[10]	Wealth quintile (least poor)	0.21 (0.09, 0.48)
	Age group (5-9 years)	16.10 (2.04, 127.20)
	Age group (10-18 years)	46.69 (6.03, 361.68)
	Age group (19-39 years)	52.42 (6.91, 397.95)
	Age group (≥40 years)	42.07 (5.41, 327.38)
	Sex (male)	1.70 (1.08, 2.66)
	Hookworm	2.78 (1.76, 4.41)
	E. coli	0.33 (0.19, 0.57)
	S. haematobium	0.12 (0.04, 0.41)
	B. hominis	1.66 (1.04, 2.67)
	P. falciparum	2.14 (1.30, 3.50)
**Soil-transmitted helminths**		
Hookworm[9]	Wealth quintile (poor)	0.34 (0.19, 0.61)
	Age group (5-9 years)	4.26 (2.08, 8.75)
	Age group (10-18 years)	7.05 (3.43, 14.49)
	Age group (19-39 years)	7.53 (3.78, 14.99)
	Age group (≥40 years)	6.48 (3.15, 13.33)
	Sex (male)	1.92 (1.39, 2.64)
	S. mansoni	2.78 (1.79, 4.31)
	B. hominis	0.70 (0.49, 0.99)
**Intestinal protozoa**		
E. histolytica/E. dispar[12,14]	*E. coli*	2.86 (1.71, 4.77)
	E. nana	1.84 (1.09, 3.08)
E. coli	*E. nana*	4.68 (3.17, 6.89)
	I. bütschlii	5.93 (3.41, 10.30)
	E. histolytica/E. dispar	3.19 (1.84, 5.55)
	S. mansoni	0.31 (0.18, 0.55)
	Age group (19-39 years)	3.63 (2.02, 6.52)
	Age group (≥40 years)	3.73 (2.00, 6.95)
	C. mesnili	3.51 (1.30, 9.48)
	Wealth quintile (very poor)	2.05 (1.16, 3.61)
	Wealth quintile (least poor)	1.91 (1.13, 3.24)
E. nana[1,5,11,15]	*E. coli*	4.88 (3.36, 7.09)
	B. hominis	2.21 (1.50, 3.23)
	E. histolytica/E. dispar	1.90 (1.12, 3.24)
	Age group (5-9 years)	4.83 (1.97, 11.81)
	Age group (10-18 years)	5.66 (2.26, 14.16)
	Age group (19-39 years)	7.52 (3.09, 18.29)
	Age group (≥40 years)	5.56 (2.19, 14.10)
I. bütschlii[5,10,14]	*E. coli*	6.56 (3.93, 10.95)
	B. hominis	2.03 (1.26, 3.28)

G. intestinalis[2,5,12]	Age group (19-39 years)	0.20 (0.08, 0.47)
	Age group (≥40 years)	0.21 (0.08, 0.58)
	I. bütschlii	0.38 (0.14, 0.99)
	S. mansoni	0.36 (0.14, 0.95)
C. mesnili[8,13,14]	*E. coli*	4.63 (1.79, 11.96)
B. hominis[5,10]	*E. nana*	2.00 (1.42, 2.83)
	I. bütschlii	2.06 (1.29, 3.29)
	S. mansoni	1.86 (1.21, 2.86)
	P. falciparum	1.39 (1.00, 1.92)

* After adjusting for: 1= *P. falciparum*, 2=*P. malariae*, 3=*S. haematobium*, 4=*S. mansoni*, 5=Hookworm, 6=*E. histolytica/E. dispar*, 7=*E. coli*, 8=*E. nana*, 9=*I. bütschlii*, 10=*G. intestinalis*, 11=*C. mesnili*, 12=*B. hominis*, 13=sex, 14=age group, 15=wealth quintile
