# Supplementary material for: The epidemiology of polyparasitism and implications for morbidity in two rural communities of Côte d’Ivoire
Source: Parasit Vectors. 2014 Feb 25;7:81. doi: 10.1186/1756-3305-7-81 (PMC3942297; doi:10.1186/1756-3305-7-81)
Supplement: Additional file 2 — Prevalence of clinical morbidity and indicators for malnutrition by sex and age group. This file can be viewed with: Adobe Acrobat reader (url: http://get.adobe.com/uk/reader/). [file 1756-3305-7-81-S2.pdf]

Additional file 2. Prevalence of clinical morbidity and indicators for malnutrition by sex and age group. 852 study participants underwent a clinical examination and had full records for anthropometric measurements for the assessment of their nutritional status.

Parameter	Overall	Sex			Age group (years)					
	pos/n (%)	Females pos/n (%)	Males pos/n (%)	p-value	<5 (n = 150)	5-9 (n = 172)	10-18 (n = 144)	19-39 (n = 237)	≥40 (n = 149)	p-value
Fever (≥38.0 °C)	43/852 (5.1)	26/431 (6.0)	17/421 (4.0)	0.184	14 (9.3)	14 (8.1)	4 (2.8)	8 (3.4)	3 (2.0)	0.005
Mean hemoglobin level (g/l)	122.4	117.9	126.4	<0.001	100.3	115.8	124.7	132.0	133.1	<0.001
Moderately anaemic	163/852 (19.1)	86/431 (20.0)	77/421 (18.3)	0.537	64 (42.7)	31 (18.0)	22 (15.3)	29 (12.2)	17 (11.4)	<0.001
Severly anaemic	3/852 (0.4)	2/431 (0.5)	1/421 (0.2)	1.000	3 (2.0)	0 (0.0)	0 (0.0)	0 (0.0)	0 (0.0)	0.015
Splenomegaly	134/852 (15.7)	59/431 (13.7)	75/421 (17.8)	0.098	60 (40.0)	48 (27.9)	13 (9.0)	6 (2.5)	7 (4.7)	<0.001
Hepatomegaly	2/852 (0.2)	0/431 (0.0)	2/421 (0.5)	0.244	0 (0.0)	1 (0.6)	0 (0.0)	0 (0.0)	1 (0.7)	0.450
Indicators of malnutrition*:										
Moderately stunted (H/A Z-score ≤2)	77/466 (16.5)	28/223 (12.6)	49/243 (20.2)	0.027	33 (22.0)	26 (15.1)	18 (12.5)	N/A	N/A	0.074
Severly stunted (H/A Z-score ≤3)	42/466 (9.0)	13/223 (5.8)	29/243 (11.9)	0.022	27 (18.0)	9 (5.2)	6 (4.2)	N/A	N/A	<0.001
Any form of stunting[#]	235/466 (50.4)	96/223 (43.1)	139/243 (57.2)	0.002	95 (63.3)	82 (47.7)	58 (40.3)	N/A	N/A	<0.001
Moderately wasted (W/H Z-score ≤2)	5/150 (3.3)	3/69 (4.4)	2/81 (2.5)	0.662	5 (3.3)	N/A	N/A	N/A	N/A	N/A
Severly wasted (W/H Z-score ≤3)	4/150 (2.7)	3/69 (4.4)	1/81 (1.2)	0.334	4(2.7)	N/A	N/A	N/A	N/A	N/A
Any form of wasting[§]	9/150 (6.0)	6/69 (8.7)	3 (3.7)	0.303	9(6.0)	N/A	N/A	N/A	N/A	N/A
Moderately underweight (W/A Z-score <2)	31/322 (9.6)	11/157 (7.0)	20/165 (12.1)	0.120	13/150 (8.7)	18/172 (10.5)	N/A	N/A	N/A	0.585
Severly underweight (W/H Z-score <3)	13/322 (4.0)	7/157 (4.5)	6/165 (3.6)	0.708	7/150 (4.7)	6/172 (3.5)	N/A	N/A	N/A	0.592
Any form of underweight[#]	124/322 (38.5)	63/157 (40.1)	61/165 (37.0)	0.561	50/150 (33.3)	74/172 (43.0)	N/A	N/A	N/A	0.075
Moderate thinness (defined by BMI)	36/852 (4.2)	19/431 (4.4)	17/421 (4.0)	0.788	5/150 (3.3)	9/172 (5.2)	9/144 (6.3)	5/237 (2.1)	8/149 (5.4)	0.259
Severe thinness (defined by BMI)	14/852 (1.6)	6/431 (1.4)	8/421 (1.)	0.560	4/150 (2.7)	1/172 (0.6)	5/144 (3.5)	0/237 (0.0)	4/149 (2.7)	0.009
Any form of thinness[#]	177/852 (20.8)	85/431 (19.7)	92/421 (21.9)	0.443	22/150 (14.7)	41/172 (23.8)	61/144 (42.4)	28/237 (11.8)	25/149 (16.8)	<0.001
Moderately malnourished (defined by MUAC)	9/536 (1.7)	4/277 (1.4)	5/259 (1.9)	0.745	8/150 (5.3)	N/A	N/A	1/237 (0.4)	0/149 (0.0)	<0.001
Severly malnourished (defined by MUAC)	1/536 (0.2)	0/277 (0.0)	1/259 (0.4)	0.483	1/150 (0.7)	N/A	N/A	0/237 (0.0)	0/149 (0.0)	0.558
Any form of malnutrition/low MUAC[#]	40/536 (7.5)	16/277 (5.8)	24/259 (9.3)	0.124	39/150 (26.0)	N/A	N/A	1/237 (0.4)	0/149 (0.0)	<0.001
Any indicator for malnutrition[§]	162/852 (19.0)	66/431 (15.3)	96/421 (22.8)	0.005	70/150 (46.7)	45/172 (26.2)	29/144 (20.1)	6/237 (2.5)	12/149 (8.1)	<0.001

* Certain nutritional indicators are applicable for specific age classes only: stunting for age group 0-14 years, wasting for children under 5 years, underweight for children up to 10 years, and MUAC is considered as an appropriate indicator for children under 5 and adults (>18 years)

[#] Of mild, moderate or severe nature

[§] Of moderate or severe nature
